# Estimating Maize Yield in the Black Soil Region of Northeast China Using Land Surface Data Assimilation: Integrating a Crop Model and Remote Sensing

**DOI:** 10.3389/fpls.2022.915109

**Published:** 2022-06-23

**Authors:** Ying Cui, Suhong Liu, Xingang Li, Hao Geng, Yun Xie, Yuhua He

**Affiliations:** ^1^Faculty of Geographical Science, Beijing Normal University, Beijing, China; ^2^Beijing Key Laboratory of Environmental Remote Sensing and Digital Cities, Beijing Normal University, Beijing, China; ^3^Land Satellite Remote Sensing Application Center, Ministry of Natural Resources of the People’s Republic of China, Beijing, China

**Keywords:** AquaCrop model, Kalman filter, remote sensing, yield prediction, random forest, data assimilation

## Abstract

Accurate yield estimation at the regional scale has always been a persistent challenge in the agricultural sector. With the vigorous emergence of remote sensing land surface observations in recent decades, data assimilation methodology has become an effective means to promote the accuracy and efficiency of yield estimation by integrating regional data and point-scale crop models. This paper focuses on the black soil area of Northeast China, a national strategic grain production base, applying the AquaCrop crop growth model to simulate the fractional vegetation cover (FVC) and maize yield from 2000 to 2020 and then forming a reliable FVC optimization dataset based on an ensemble Kalman filter (EnKF) assimilation algorithm with remote sensing products. Using the random forest model, the regression relationship between FVC and yield was established from the long-term time series data, which is crucial to achieve better yield estimation through the optimized FVC. The major findings include the following: (1) The R^2^ of the assimilated FVC and maize yield can reach 0.557. (2) When compared with the local statistical yield, our method reduced the mean absolute error (MAE) from 1.164 ton/ha (based on GLASS FVC products) to 1.004 ton/ha (based on the calibrated AquaCrop model) and then to 0.888 ton/ha (the result after assimilation). The above results show that we have proposed a yield estimation method to provide accurate yield estimations by combining data assimilation and machine learning. This study provided deep insights into understanding the variations in FVC and revealed the spatially explicit yield prediction ability from the time series land surface parameters, which has significant potential for optimizing water and soil resource management.

## Introduction

Agricultural production is the foundation of human survival. Accurate prediction of crop yield enables farmers to estimate profits and adjust crop-planting patterns. Governments also benefit from yield prediction information, which helps to promote the development and reform of food security, sustainable utilization of water and soil resources, agricultural trade and many other aspects (Feng et al., 2021).

The northeastern Chinese black soil area is a major black soil zone of the world and a strategic guarantee base for national food security by producing crops such as maize and soybean (Lin et al., 2019). The special soil properties and natural geographical conditions make its rain-fed agricultural production mode different from other main grain-producing areas. Atmospheric precipitation has become one of the main influencing factors of crop yield (Cui et al., 2021). According to these characteristics of this area, there is an urgent need to carry out detailed research on crop yield estimation, especially on maize with large planting scale and appreciable economic benefits (Qian et al., 2018).

Crop growth models are an important tool to quantitatively describe the growth and yield formation process of specific crops under specific environments based on point scales (Van et al., 1989; Monteith, 1996; Steduto et al., 2009), and it is difficult to characterize the spatial low of crop growth due to the limitation of scale. The current research trend is to run models on a regional scale and then establish the response system of crop growth to soil and meteorological environment changes to provide macro decision-making for precision agriculture. Different from photosynthetic effective radiation driving mechanism of WOFOST model and the other crop model (Todorovic et al., 2009), AquaCrop model is driven by water factors and simulates crop yield based on water use efficiency (Raes et al., 2009) and is widely used in arid and rain-fed areas with great research prospects in water-efficient utilization (Eshete et al., 2020). This model evolved from the crop water response equation in the irrigation and drainage Document No. 33 of the Food and Agriculture Organization of the United Nations (FAO) (Passioura, 1996). Several studies have applied AquaCrop model to simulate and evaluate typical crops growth in different regions and got great results. Iqbal et al. (2014) demonstrated that the yield of winter wheat in North China Plain simulated by AquaCrop model performed unsatisfied under strong water stress, and the accuracy is significantly improved after parameter calibration. Sandhu and Irmak (2019) found the AquaCrop simulated yield and evapotranspiration quite well in Midwestern America, but encountered substantial difficulties in simulating biomass and soil-water. The AquaCrop model driven by water has advantages for crop research in Northeast China, but relevant research has lacked.

Simulation and observation are two basic strategies of geoscience research (Li, 2016). Since almost all surface variables have high spatiotemporal heterogeneity, there is bound to be deviation and uncertainty in any parameterization process. From the perspective of simulation, although the crop model has sorted out the material cycle and other growth processes very clearly on the micro level, complex error transmission will accumulate, and the uncertainty reflected will be amplified in the simulation results when it is extended to the macro scale. From the perspective of observations, field measurements have high accuracy but low spatial representation. Obtaining abundant observations will be very laborious when studying a large-scale area with high heterogeneity. Although remote sensing observations already provide sufficient surface data, a sophisticated inversion algorithm is needed to gain effective information indirectly. The inversion and sensor accuracy greatly limit the reliability of remote sensing data. Therefore, integrating multisource observations and decreasing the uncertainty of the simulation is key to ameliorating the limitations of model application.

Data assimilation (DA) is an important integrating methodology that automatically adjusts the process model forward direction continuously by relying on observations and then generates the minimum-deviation state variable set with spatiotemporal consistency, which is usually used for modeling and dynamic prediction of complex systems (Li and Bai, 2010; Qin et al., 2018). With the rapid development of remote sensing technology, a large amount of earth observation data has emerged as crucial support for regional precision agriculture research. By reanalyzing the prior crop growth model with multi-temporal observation data, the reliability of the simulation results is improved, which is of great significance to agricultural dynamic monitoring, yield prediction, and regional resource management.

Looking at the relevant studies integrating crop model and remote sensing data in recent years (Table 1), the most common crop models are WOFOST, CERES and AquaCrop. The main crop types are winter wheat and other food crops. Optical remote sensing data is generally used for remote sensing observation data, and a few literatures show that InSAR has a good effect on retrieving soil water content (Wigneron et al., 2017). The assimilation variable generally selects LAI (leaf area index) for various crop models, while there are different options for AquaCrop model. In terms of assimilation algorithm, both cost function algorithm and filtering algorithm have more applications and innovations. The research objectives mostly focus on improving the accuracy of yield estimation. Most of the available studies using short-term data, mostly less than three growing seasons and may neglect the inter-annual variability in environmental conditions.

**TABLE 1:** Research status of the crop model and remote sensing data assimilation.

Authors	Model	Crop	Data	Variable	Method	Objective	Region	Period
Hank et al., 2015	PROMET	Winter wheat	Landsat-TM; RapidEye	LAI	Filter	Regional yield estimation	Germany	2010-2011
Jin X. et al., 2016	AquaCrop	Winter wheat	Hyperspectral	AGB	PSO	Yield estimation	Beijing, China	2008-2011
Jin H. et al., 2016	CERES-Maize	Maize	MOD09A1	LAI	SA	LAI estimation	Jilin, China	2010
Xie et al., 2016	CERES-Wheat	Winter wheat	Landsat	LAI; SWC; AGB	PF	Yield estimation	Shanxi, China	2007-2014
Iqbal et al., 2014	AquaCrop; SAFY	Wheat	HJ-1; Landsat-8	LAI; CC	EnKF; PSO	Yield estimation	Yangling, China	2012-2015
Li et al., 2017	CERES-Wheat	Winter wheat	GF-1; HJ-1; Landsat-8	LAI	PF; 4DVar	Yield estimation	Hebei, China	2014
Gilardelli et al., 2019	WARM	Rice	Landsat-7/8; Sentinel-2	LAI	Downhill simplex method	Yield estimation	Italy	2014-2016
Hu et al., 2019	SWAP-WOFOST	Sugarcane	(Field experiment)	LAI; SWC	Forcing method; calibration method; EnKF	Yield estimation	Guangxi, China	2016-2017
Li et al., 2019	WheatSM	Winter wheat	MCD15A3; MCD15A3H	LAI	EnKF; SCE-UA	Yield estimation	Henan, China	2013-2017
Pique et al., 2020	SAFY-CO_2_	Winter Wheat	Sentinel-2; SPOT-2/4/5	GAI	SAFY-CO_2_	Assessing annual carbon budget	France	2005-2014
Tewes et al., 2020	LINTUL5	Winter Wheat	(Field experiment)	LAI	EnKF; WM	Yield estimation	France; Germany; Netherlands	2016-2017
Wu et al., 2021	WOFOST	Winter wheat	HJ-1; GF-1	LAI	ABT-4DVar	Yield estimation	Hebei, China	2013-2014
Peng et al., 2021	SAFY	Maize	UAV	LAI	EnKF	Yield estimation	Inner Mongolia, China	2019

*AGB: Above ground biomass. CC: Canopy cover. PSO: Particle swarm optimization. SA: Simulated annealing. SCE-UA: Shuffled complex evolution. LAI: leaf area index. GAI: Green area index. UAV: unmanned aerial vehicle. LSOA: Least squares optimization algorithm. SWC: Soil water content. PF: particle filter. WM: Weighted Mean.*

Although many studies have indicated the effectiveness of using crop models and remote sensing data assimilation for yield mapping, the expansion accuracy from the field to regional scale still lacks exploration (Steele-Dunne et al., 2017). Silvestro et al. (2017) used the updating assimilation method, the ensemble Kalman filter (EnKF), assimilated leaf area index (LAI) into the SAFY model, and used the calibration assimilation method, PSO, and assimilated canopy cover (CC) into the AquaCrop model to demonstrate the possibility of estimating wheat yield. The results show that the relative root-mean-square error (RRMSE) between the predicted and the measured yield ranges from 0.18 to 0.24 t/ha. SAFY with the EnKF method was more suitable than Aquacrop with PSO, which is mainly due to the high computational cost and the difficult calibration of the AquaCrop model. For the AquaCrop model, CC achieved a lower RMSE than LAI. Another finding of this article is that the accuracy of the assimilation method is greatly limited by the number of remote sensing images, three or four images with an error in LAI estimation of 30% and an error in the yield estimation of approximately 18%. Feng et al. (2019) used the multivariable linear regression model (MLR) and the RF model as external modifications of the APSIM crop model to predict wheat yield, while RF has a higher accuracy gain than MLR. The R^2^ and root-mean-square error (RMSE) between the predicted yield and the measured yield were 0.81 and 0.54 t/ha and 0.61 and 0.86 t/ha, respectively, before and after combining RF. Hu et al. (2019) used forcing, calibration, and EnKF, three assimilation methods for improving sugarcane crop simulation. The results show that EnKF performed the best in estimating soil water content, LAI development, and sugarcane yield. Assimilating LAI alone works better than assimilating LAI and SWC both under slight water stress levels, which demonstrates that the choice of assimilated variable relies on a reasonable diagnosis of the environment. In summary, the direct assimilation of remote sensing data to update vegetation assessments is very promising. Very limited literature available in Northeast China for yield estimation using data assimilation, especially for long-term study.

This study completed the calibration and validation of the AquaCrop model in Northeast China via field test data and applied 21 years of regional-scale simulations from 2000 to 2020. Then we selected FVC for AquaCrop model assimilation not only because it is one of the outputs of AquaCrop but also because it considers the important role it plays in surface process simulation. Due to the accumulation of sufficient assimilated FVC data and crop yield data, which reflect various environmental conditions, the relationship between yield and environmental factors was established through the machine learning method of random forest. Relying on the FVC assimilation curve, a high-precision estimation of crop yield can be obtained. This study aims to establish an assimilation system to better monitor FVC growth and provide better maize yield estimation, which is significant for local agricultural management.

## Materials and Methods

### Study Area

**FIGURE 1:**
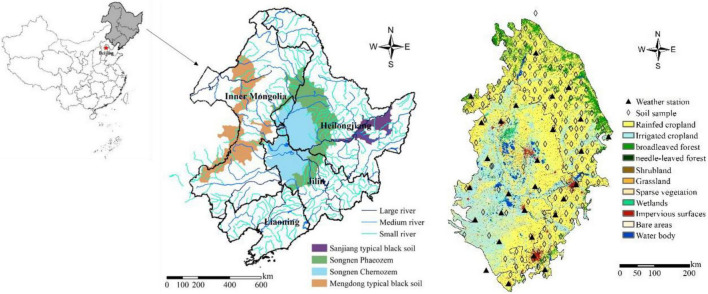
Distribution of phaeozem, chernozem and rivers in Northeast China **(left)**; distribution of 30 m-resolution land cover type, meteorological stations and soil sampling points in Songnen Plain in 2020 **(right)**.

The Songnen Plain (42°56′ ∼ 50°03′ N, 122°05′ ∼ 128°12′ E) is located in the middle of the Songliao black soil basin in Northeast China, with a total area of approximately 206404.3 km^2^ and a large part of it is dominated by rain-fed agriculture. It has a temperate continental monsoon climate, the average annual temperature is approximately 0.4°C, the average annual precipitation is approximately 534 mm, and the altitude is approximately 250∼450 m. In 2021 Liu et al. (2021) classified the phaeozem and chernozem as typical black soil that is covered with a high content of organic matter dark humus and mapped three typical black soil areas in Northeast China which are Sanjiang, Songnen and Mengdong. This study took the Songnen typical black soil region as the study area, composed of Songnen Phaeozem region and Songnen chernozem region. Most farmland in the study area was planted with soybeans and maize without irrigation scheduling. The irrigation scenario was not set up in the subsequent application of crop model. This paper established a database from 28 meteorological stations and 155 practical soil profile samples, scattered as shown in Figure 1. The phenological information of maize is shown in Table 2.

**TABLE 2:** Phenological information of maize in the black soil area of Northeast China.

Growth Stages	Length days	Date
Sowing	1	1 May
Emergence	6	7 May
Maximum canopy cover	54	24 June
Maximum rooting depth	108	17 August
Start of canopy senescence	107	16 August
Maturity	150	27 September
Flowering	66 – 79	6 July – 19 July
Yield formation	66 – 150	6 July – 27 September

### Meteorological Data

This study collected meteorological data from 28 weather stations from 2000 to 2020, including daily rainfall (mm), minimum and maximum temperature (°C) and solar radiation (MJ m^–2^ day^–1^), as input to the AquaCrop meteorological module (see Appendix A for details). These data can be downloaded from National Meteorological Information Center^1^. Meanwhile, the reference evapotranspiration is calculated through the FAO ET_0_ calculator.

### Soil Sampling

**FIGURE 2:**
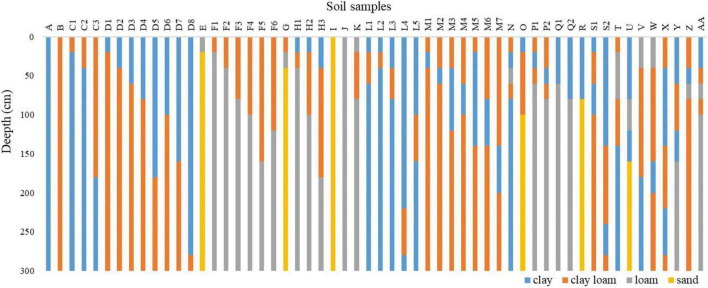
Detailed types of soil profile in Songnen Plain (soil depth: 0-300 cm).

Soil properties have a major impact on crop yield chiefly because they affect the ability of soil to retain water and transfer water to crops (Bakker et al., 2007). AquaCrop model provides reference soil parameters for all kinds of soil texture, which reduces the difficulty of regional soil-property-information measurement. Therefore, a large number of measured soil texture data are used for establishing the corresponding soil module parameters of the AquaCrop model to ensure that the model considers the impact of soil hydraulic properties of different soil texture structures on crop growth. This study matched the nearest meteorological station data for 155 soil survey points by spatial location, and conducts regional AquaCrop simulations on these 155 units. The soil texture of 155 survey points in the Songnen black soil area is classified into 27 categories and can be further divided into 56 subcategories according to the thickness of each layer (Figure 2). The AquaCrop model assigns 56 groups of parameters in soil module according to these soil testure subcategories. The classification results show the high soil heterogeneity in Songnen Plain, although there are only four different textures. Soil classification improves the efficiency of regional model operation.

### Satellite-Based Data

GLASS fractional vegetation cover (FVC) products from 2000 to 2020 were collected for regional data assimilation. Multisource high-resolution optical remote sensing images in 2018 and 2020 were collected for relative accuracy verification of the GLASS FVC product and assimilation tests.

GLASS FVC is a global fractional vegetation cover product, with 500 meters spatial resolution and 8 days temporal resolution, published by the National Earth System Science Data Center^2^. The inversion algorithm is based on multisource remote sensing data and measured site data (Jia et al., 2015). This study adopted the GLASS FVC data with tile number h26v04 from 2000 to 2020.

Nenjiang is located at 49°10′ N, 125°13′ E, altitude of 242.2 meters, and Lishu is located at 43°10′ N, 124°19′ E, altitude of 165.7 meters. These two counties are the northernmost and southernmost parts of the Songnen black soil area, respectively. This study collected multiscene domestic GaoFen-1 satellite remote sensing images of Nenjiang County, Heilongjiang Province, in 2018 and Lishu County, Jilin Province, in 2020 with 8-meter spatial resolution and 4-day temporal resolution. Several domestic ZiYuan-3 satellite images and Landsat-8 images were collected as supplements with 6-meter spatial resolution and 5-day temporal resolution and 30-meter spatial resolution and 16-day temporal resolution (Table 3). The absolute calibration coefficient and solar irradiance are from the official website of the China Resources Satellite Application Center^3^.

**TABLE 3:** List of satellite imagery collected in Nenjiang County and Lishu County.

Area	Data	Satellite	Sensor	Path/Row	Area	Data	Satellite	Sensor	Path/Row
LS	2020-04-04	GF-6	WFV	589/63	NJ	2018-05-05	LS-8	OLI	120/26
LS	2020-05-28	GF-6	WFV	594/63	NJ	2018-05-07	GF-1	PMS2	597/72
LS	2020-06-12	GF-1	WFV4	601/81	NJ	2018-06-21	GF-1	PMS1	597/72
LS	2020-06-28	GF-1	WFV3	597/81	NJ	2018-06-21	ZY-3-2	TMS	884/102
LS	2020-07-15	GF-1	WFV4	603/81	NJ	2018-07-27	GF-2	PMS1	1004/103
LS	2020-07-23	GF-1	WFV3	600/81	NJ	2018-07-27	GF-2	PMS2	1003/113
LS	2020-07-23	GF-1	WFV4	600/81	NJ	2018-08-01	GF-1	WFV3	596/72
LS	2020-08-16	GF-1	WFV3	596/81	NJ	2018-08-05	GF-1	WFV2	598/72
LS	2020-08-21	GF-1	WFV4	601/81	NJ	2018-08-18	GF-1	WFV3	598/72
LS	2020-09-06	GF-1	WFV3	600/81	NJ	2018-08-18	LS-8	OLI	119/26
LS	2020-09-26	GF-1	WFV2	595/81	NJ	2018-08-22	GF-1	WFV3	599/72
LS	2020-09-26	GF-1	WFV3	595/81	NJ	2018-09-10	ZY-3	MUX	884/102
LS	2020-10-13	GF-1	WFV4	601/81	NJ	2018-09-15	ZY-3	MUX	883/101
LS	2020-10-27	GF-6	WFV	603/60	NJ	2018-10-17	ZY-3-2	TMS	884/102
LS	2020-11-05	GF-6	WFV	594/63	NJ	2018-10-18	GF-2	PMS1	1004/113
LS	2020-11-08	GF-1D	PMS	654/82	NJ	2018-10-18	GF-2	PMS2	1004/112
LS	2020-11-13	GF-1B	PMS	653/82					

After geometric correction and radiometric correction, the fractional vegetation cover (FVC) can be retrieved according to the pixel dichotomy:


(1) $$\text{FVC}=\frac{\mathrm{VI}-{\text{V}}_{\text{s}}}{{\text{V}}_{\text{v}}-{\text{V}}_{\text{s}}}$$

where VI is the NDVI of the pixel, V_s_ is the NDVI of the pure soil pixel, and V_v_ is the NDVI of the pure vegetation pixel.

**FIGURE 3:**
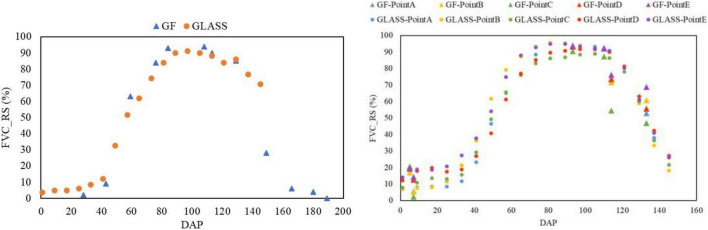
Verifying GLASS FVC with GF satellite image inversion. Lishu County in 2020 **(left)**; Nenjiang County in 2018 **(right)**.

The remote sensing FVC curves obtained from different sensors and inversion methods are roughly the same, reflecting the reliability of the GLASS FVC product in Northeast China. Figure 3 shows the time sequence FVC of the two main maize-producing areas on the Songnen Plain. In the early stage of maize growth, the FVC was almost less than 0.15. From approximately 40 to 80 days, the FVC in most areas increased rapidly from 0.20 to 0.9. After approximately 120 days, FVC reached a high level, and maize started to mature. Some leaves began to curl, and those at the lower part of the canopy began to turn yellow. FVC showed a decreasing trend. By approximately 140 days, the FVC decreased to 0.6, and maize was ready to mature. By approximately 150 days, maize was harvested, and the FVC was below 0.2. The surface of black land was mainly cut straw.

### Statistical Yield

**FIGURE 4:**
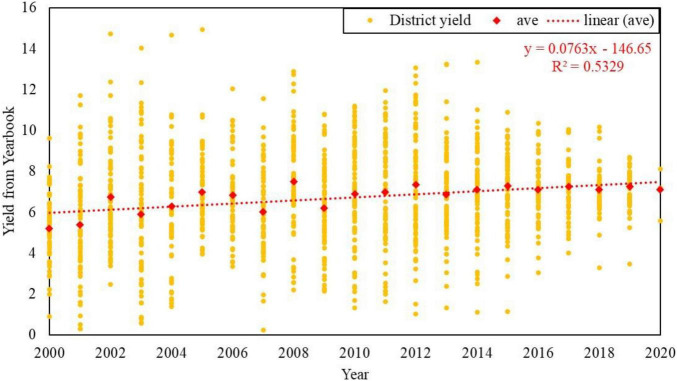
Maize yield (t/ha) dataset from 2000 to 2020’s Statistical Yearbook.

National statistical yearbooks are formed by governments’ sampling surveys and have acknowledged the authenticity. This study collected province yearbooks from 2000 to 2020 for Jilin, Heilongjiang, Liaoning and Inner Mongolia, which are under the coverage of the Songnen Plain. The established yield dataset of Northeast China included 3666 statistical maize yields per unit area of 258 districts and counties, which was used to establish the regression relationship between FVC and yield based on spatial location. The linear fitting results of yearly yield are shown in Figure 4, which need “detrending” to eliminate the impact of these unquantifiable factors on the simulation accuracy (Wang X. et al., 2020), considering the progress of corn varieties and planting technology over the past 21 years while we applied field management measures in 2018.

## Models and Methods

**FIGURE 5:**
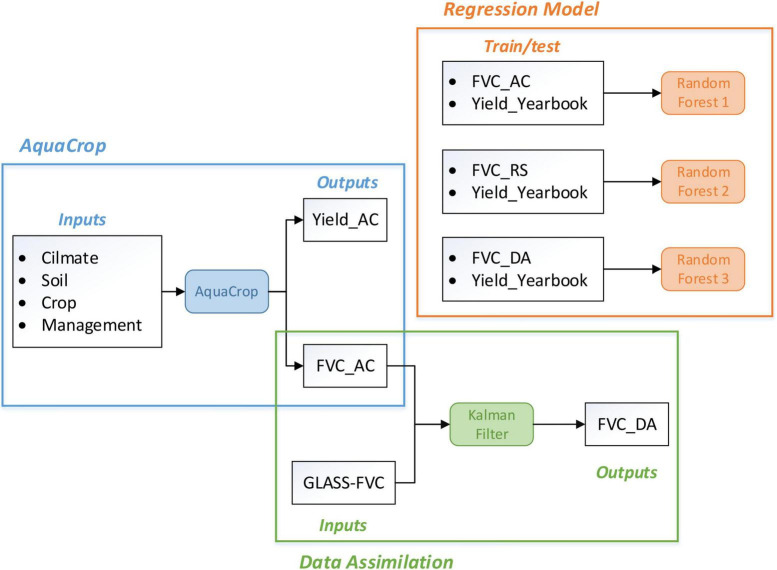
Flowchart of the methodology applied.

The methodology adopted in this study mainly involved three parts: regional application of the AquaCrop model, FVC data assimilation by model outputs and remote sensing products, and yield estimation relying on the RF regression model (Figure 5).

### AquaCrop Model

The AquaCrop model simulates the transpiration process of crops by inputting climate, crop, soil, and field management data and finally outputs the daily prediction results of CC, rooting depth, biomass and yield (Raes et al., 2009; Steduto et al., 2009). A significant difference from other crop models is that AquaCrop uses CC instead of LAI as the basis for calculating transpiration, separates soil evaporation from leaf transpiration and avoids the confusion effect of unproductive water consumption (Equations 2-3). This variable obviously simplifies the simulation and integrates the leaf expansion growth, angle and distribution into an overall growth function. Another advantage is that CC can be easily obtained from remote sensing sources to check analog CC or as input of AquaCrop. Equation 4 and Equation 5 are the two stages of CC growth and the stage of CC decline, respectively.


(2) $$\text{Tr}={\text{Kc}}_{\text{Tr}}\times {\text{ET}}_{0}$$

(3) KcTr=CC*×KcTr,x

where Tr is the leaf transpiration. Kc_Tr_ is the crop transpiration coefficient. ET_0_ is the relative evapotranspiration without stress. CC^*^ is the adjusted canopy cover. Kc_Tr,x_ is the coefficient for maximum crop transpiration, which represents an integration of the effects of the characteristics that distinguish the crop with a complete canopy from reference grass.


(4) $$\left\{ \begin{array}{c} \text{CC}={\text{CC}}_{0}\times {\text{e}}^{\text{CGC}\times \text{t}},\text{CC}\leq \frac{{\text{CC}}_{\text{x}}}{2}\\ \text{CC}={\text{CC}}_{\text{x}}-\left({\text{CC}}_{\text{x}}-{\text{CC}}_{0}\right)\times {\text{e}}^{-\mathrm{CGC}\times t},\text{CC}>\frac{{\text{CC}}_{\text{x}}}{2} \end{array}\right.$$

(5) $$CC={\text{CC}}_{\text{x}}\left[\begin{array}{c} 1-0.05\left(\begin{array}{c} {\text{exp}}^{\frac{\text{CDC}}{{\text{CC}}_{\text{x}}}} \end{array}\text{t}-1\right) \end{array}\right]$$

CC is canopy coverage (%). t is the time accumulated from emergence. CC_0_ is the initial canopy coverage (%), generally taking the average seedling coverage at 90% emergence. CC_x_ is the maximum value of canopy coverage (%). CGC is the canopy coverage growth rate, which indicates the increase in canopy coverage per unit growth degree day (% GDD^–1^). CDC is the canopy coverage decrease rate, which represents the reduction in daily canopy coverage per unit growth degree day (% GDD^–1^).

The accumulated biomass is calculated by daily transpiration (Tr_i_), daily atmospheric evapotranspiration demand (ET_0,i_) and normalized water productivity (WP^*^), and crop yield is calculated by the biomass and harvest index, as shown in Equations 6-7.


(6) $${\text{B}}_{\text{i}}={\text{WP}}^{*}\times \left(\frac{{\text{Tr}}_{\text{i}}}{{\text{ET}}_{0,i}}\right)$$

(7) Y=B×HI

A soil database can be established according to the layered structure of soil texture in the AquaCrop model, which covers 13 soil types (Table 4). The 155 measured soil texture data in Songnen black soil region can be flexibly set to found 57 groups of the corresponding parameters based on this table.

**TABLE 4:** AquaCrop model soil database (Van Gaelen and Raes, 2016).

Soil tape	TAW (mm/m)	PWP (vol%)	FC (vol%)	SAT (vol%)	Ksat (mm/day)	tau	CN	REW (mm)
Sand	70	6.0	13.0	36.0	3,000.0	1.00	46A	4
loamy sand	80	8.0	16.0	38.0	2,200.0	1.00	46A	5
sandy loam	120	10.0	22.0	41.0	1,200.0	1.00	46A	7
Loam	160	15.0	31.0	46.0	500.0	0.76	61B	9
silt loam	200	13.0	33.0	46.0	575.0	0.80	61B	11
Silt	240	9.0	33.0	43.0	500.0	0.76	61B	11
sandy clay loam	120	20.0	32.0	47.0	225.0	0.58	72C	9
clay loam	160	23.0	39.0	50.0	125.0	0.47	72C	11
silty clay loam	210	23.0	44.0	52.0	150.0	0.50	72C	13
sandy clay	120	27.0	39.0	50.0	35.0	0.30	77D	10
silty clay	180	32.0	50.0	54.0	100.0	0.43	72C	14
Clay	150	39.0	54.0	55.0	35.0	0.30	77D	14

*Thickness is set to 4.00 m in the model. PWP: permanent wilting point. FC: field capacity. CN: Curve Number. REW: Readily Evaporable Water.*

After the sensitivity analyzed and parameters calibrated in the Hebei basin of Nenjiang County based on field experiments from 2011-2018, AquaCrop model has a good prediction effect on a point scale in Northeast China (Xie et al., 2003; Cui et al., 2021). Present research carry on using the same set of cultivar parameters from the previous study (see Appendix B for details).

### Sequential Filter Assimilation

Referring to the application summary of remote sensing and crop model assimilation, modern data assimilation methods are mainly divided into the following two categories: parameter optimization methods based on cost functions and sequential filtering methods based on estimation theory (Huang et al., 2018). The former minimizes the difference between the remote sensing observation value and the model simulation value using iterations to achieve the optimal estimation, while the latter constantly relies on external observations to adjust the model simulation trajectory in real time to achieve the optimal prediction. For the yield estimation proposition, filter assimilation is more widely used because of its real-time performance. The most representative method is the EnKF, which is based on linear and Gaussian assumptions. In the context that the AquaCrop model simulated a daily FVC curve in the growing season and the GLASS FVC also provides multitemporal FVC values, it is possible to make FVC as the assimilation variable attempt to obtain a higher accuracy estimation based on ensemble Kalman filter (EnKF) algorithm.

KF is an assimilation algorithm that uses the linear system state equation to estimate the optimal system state through the system input and output observation data. Since the observation data include the influence of noise and interference in the system, the optimal estimation can also be regarded as a filtering process. The AquaCrop simulated FVC_AC and remote sensing inversion calculated FVC_RS contribute to the following dynamic models, which are constructed to evolve FVC in time and used to provide short-range prediction of FVC:


(8) $${\text{FVC}}_{\text{t}}={\text{F}}_{\text{t}}\times {\text{FVC}}_{t-1}+w_{t}$$

(9) $$w_{t}={\text{FVC\_RS}}_{\text{t}}-{\text{FVC\_AC}}_{\text{t}}$$

where FVC_t−1_ is the FVC value of the previous time, FVC_t_ represents the present FVC value, *w_t* is the model error. FVC_RS_t_ represents the FVC data observated from GLASS FVC products at time t. FVC_AC_t_ represents the FVC data simulated by the AquaCrop model at time t, which is from the daily dataset during the crop growth period. F_t_ is a linear state transition operator:


(10) $${\text{F}}_{\text{t}}=1+\frac{1}{\left|{\text{FVC\_AC}}_{\text{t}}+\varepsilon \right|}\times \frac{{\text{dFVC\_AC}}_{\text{t}}}{\text{dt}}$$

where is an uncertainty factor that is set to 0.0001 to prevent the denominator from being 0. The observation operator is set to 1 due to the same parameter of simulation and observation.

EnKF integrates the model forward with the new observation data to obtain a set of analysis field sets, which are updated by the KF equation; the updated set, as the background field of the next moment, continues to make forward short-term forecasts and is assimilated with the new observation data of the next moment. EnKF overcomes the weakness that KF is limited to dealing with linear problems and solves the problem caused by the KF method requiring too much computing resources when calculating the covariance of prediction error. For KF assimilation, the observation operator H in EnKF is set to 1, but the process model error is set to 5% of the predicted value, the observation error is set to 5%, and the number of set elements is set to 20, 100, and 200.

### Random Forest

In order to extend the improvement of FVC accuracy to yield estimation accuracy, this section build a regression model of time series FVC and maize yield in a data-driven approach. Machine learning is currently the most effective and rapidly developing data research method, which enable algorithmic models to learn knowledge from data autonomously and then have the ability of judge and predict in new problems. Random forest (RF) is a typical classifier that uses multiple decision trees to train and predict from input samples. It determines the category of test samples by voting and then takes the average output of each decision tree as the final result.

Referring to the Scikit-learn python machine learning library, this study uses the daily FVC value to estimate yield optimally through the RF model. In order to compare the impact on yield estimation accuracy before and after FVC assimilation, three random forest models were established to describe the regression relationship between the FVC curve and yield. The difference is in the input FVC, including the FVC simulated by AquaCrop, the FVC provided by the GLASS remote sensing product and the FVC obtained by data assimilation. Finally, the model with the highest accuracy is selected for maize yield prediction, so as to achieve the research goal of optimal yield estimation.

The total dataset of RF1-3 is composed of 21 growing seasons from 2000 to 2020, three types of FVC data of 155 simulation units and the corresponding statistical yield data. Each RF model should have 3255 records, each record have a set of FVC and a statistical yield. The actual number of records is finally determined by the amount of data with well assimilation effect. The scale of statistical yearbook data is suitable, which is of 258 districts and counties can be matched with the three series of FVC data on 155 simulation units. Another consideration of choosing statistical yield instead of AquaCrop simulated yield is to avoid the accuracy interference caused by the same model output as FVC_AC, and more objectively reflect the real harvest. The samples were randomly divided into two parts with a ratio of 8:2 the large part used for training and the other part used for accuracy evaluation (Table 5). The accuracy verification is indicated by MAE (mean absolute error) and R^2^, using Equations 11-12.


(11) $$\text{MAE}=\frac{1}{m}\sum_{i=1}^{m}\left|\left(y_{i}\begin{array}{c} - \end{array}\hat{y_{i}}\right)\right|$$

(12) $$R^{2}=1-\frac{\sum {\left(y_{i}\begin{array}{c} - \end{array}\hat{y_{i}}\right)}^{2}}{\sum {\left(y_{i}\begin{array}{c} - \end{array}\bar{y}\right)}^{2}}$$

**TABLE 5:** Data composition of the random forest model.

Name	Year	Area	Input (FVC)	FVC frequency	Output (Yield)
RF1	2000-2020	Songnen typical black soil region	FVC_AC	150	Y_ yearbook
RF2	2000-2020	Songnen typical black soil region	FVC_RS	17	Y_yearbook
RF3	2000-2020	Songnen typical black soil region	FVC_DA	150	Y_yearbook

*FVC_AC is the FVC simulated by the AquaCrop model. FVC_RS is the GLASS FVC data. FVC_DA is the assimilation result. The FVC frequency shows the FVC number of a data array. The AquaCrop model gives daily output during the 150-day growth period. GLASS FVC provides observations every 8 days.*

## Results and Analysis

### Regional Continuous Simulation of the AquaCrop Model

**FIGURE 6:**
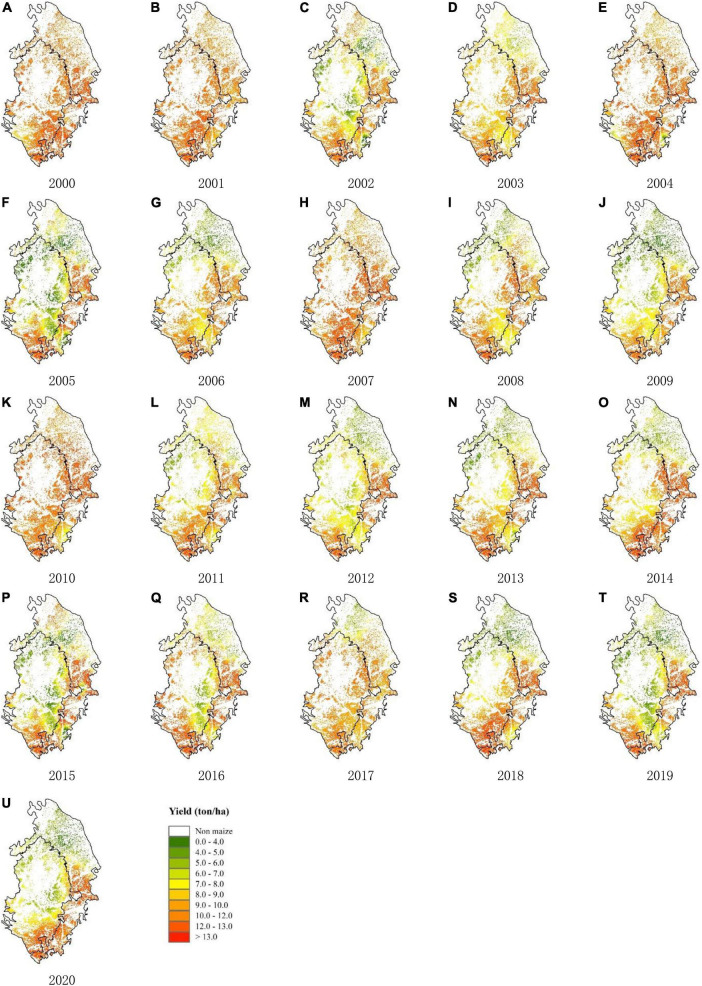
Maize yield map (ton/ha) for Songnen Plain simulated by AquaCrop model from 2000–2020.

Running the AquaCrop model of multithread scheduling for the 155 soil representative cells in Songnen black soil region from 2000 to 2020, we explored the spatial pattern of predicted yield by mapping the results after two empirical Bayesian Kriging interpolation to Figure 6.

**FIGURE 7:**
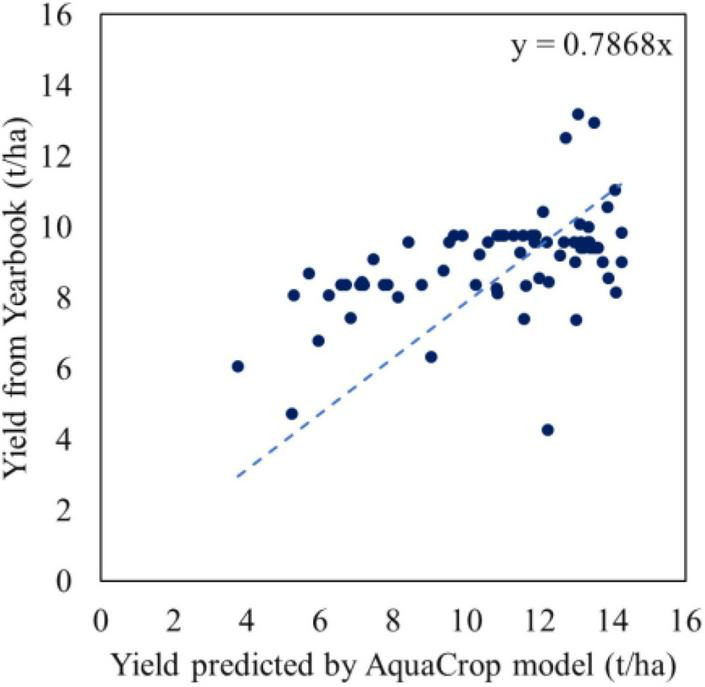
Comparison of random AquaCrop simulated yield and the corresponding statistical yield.

In general, the spatial distribution and the temporal differences of predicted yield are reasonable reproduced, which is consistent with that of statistical yield (shown in Figure 7). Among them, the output of the southeast is the highest, followed by the southwest, and the northeast is the lowest. This spatial pattern is mainly caused by temperature and soil texture. During the growing season, the northern part of the study area is approximately 5°C lower than the southern part as a whole, as shown in Appendix A. A lower temperature makes it more difficult for crops to reach the effective accumulated temperature required for maturity, resulting in a lower final yield. Regarding the soil texture difference, although the fertility of chernozem is higher than that of phaeozem, it requires supplementary irrigation to effectively improve growth due to its relatively poor water storage capacity. For Northeast China, which relies on precipitation to provide water supply, phaeozem is more suitable for cultivated land than chernozem.

In terms of time series, the inter-annual differences were mainly caused by the differences of hydrothermal conditions in each growing season, especially the water factor. The output showed a stable trend, with 2000, 2001, 2004, 2007, 2010, 2017 being the good harvest years for the entire region, while other years clearly make out the spatial difference. Abnormal precipitation events during crop growth period, especially in the process of yield formation after flowering, are easy to cause low crop yields. Mild and continuous natural precipitation provides optimal water conditions for crop growth and development. The neglected soil erosion and the administration policies also affect the estimation accuracy.

### Field Scale Assimilation of Fractional Vegetation Cover

**FIGURE 8:**
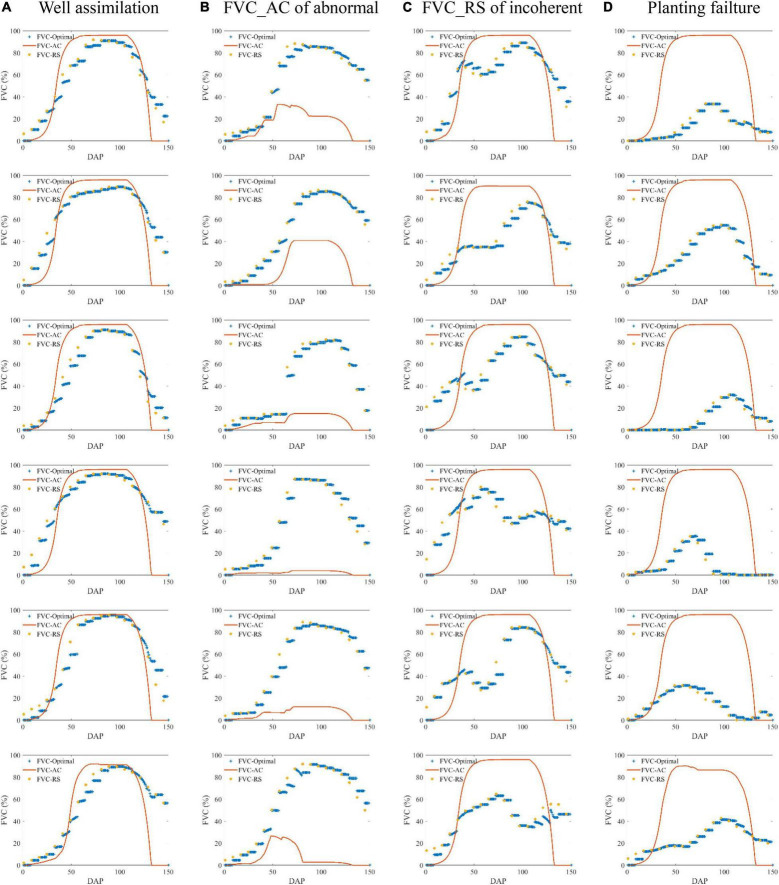
Several examples of FVC assimilation based on the EnKF multithreaded algorithm.

This study implements a multithreaded univariate EnKF assimilation algorithm based on MATLAB language, which can realize the optimal estimation combining the daily FVC simulated by the AquaCrop model and the multitemporal remote sensing FVC in the corresponding growing season. The results show that EnKF assimilation can indeed integrate remote sensing data into simulation results, achieve higher spatiotemporal resolution and provide FVC estimation with good accuracy. All the assimilation results are defined into 4 types (Figure 8). Including: a) Approximately 80% of FVC_Optimal showed excellent assimilation results due to the similar shape of FVC_AC and FVC_RS. b) Approximately 1% of FVC_Optimal showed poor performance owing to the abnormal maize canopy growth reflected by FVC_AC, which differed greatly from the remote sensing observations at these points. c) A few FVC_RS fails to maintain coherence and showed the FVC inversion results of some phases may be distorted, which lead to doubts about the reliability of FVC_Optimal after correction according to the observed value. d) Approximately 10% of FVC_RS is generally lower than the corresponding FVC_AC, indicating an obvious failure to achieve the desired high level in canopy growth. It can be considered that maize planting failed at these locations.

The unsatisfactory assimilation results were probably caused by mixed-pixel RS inversion, spatial mismatch and inaccurate phenological parameters that led to discrepancies between the simulation and reality. It should be noted that remote sensing data can reflect real surface information only when the spatial resolution is sufficiently high. The accuracy of the method will be dramatically promoted when the resolution of remote sensing input data is improved.

At the end of the growth period, FVC observed by remote sensing is not of maize crops, but background interference such as surface weeds, it will have no impact on yield simulation by removing the last 10 days of data before adding to the RF model.

### Random Forest Model of Regression Between Fractional Vegetation Cover and Yield

**FIGURE 9:**
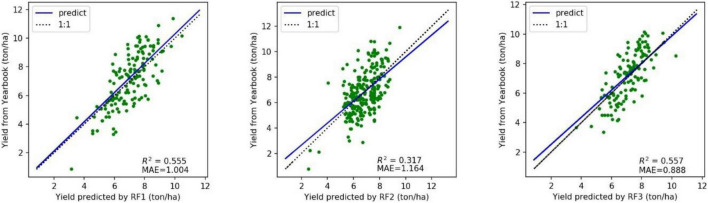
Comparison of accuracy between the three random forest models. (RF1: input FVC_AC; RF2: input FVC_RS; RF1: input FVC_Optimal).

Based on the FVC array and yield data in the last 20 years, this study established three RF models to describe the regression relationship between the three FVCs and the yield of the statistical yearbook. RF1 used the FVC simulated by the AquaCrop model, RF2 used the remote sensing GLASS FVC product, and RF3 used the optimal FVC assimilated by the two. Considering that the unsatisfactory assimilation results reflected the unreliability of FVC_AC or FVC_RS, the three random forest models only used the data corresponding to well-assimilated fraction of FVC in Figure 8. The verification results of the predicted yields of random forests are shown in Figure 9. As a typical data-driven machine learning model, random forest strongly shows the dependence of prediction results on input data, and they objectively reflect the invisible relationship between FVC and yield.

Based on the optimal FVC curve, RF3 showed the best performance on maize yield prediction in the black soil area of Northeast China. RF3 is slightly better than RF1, which proves that the addition of remote sensing observation data still has a gain on the yield simulation model, R^2^ increases from 0.555 to 0.557, and MAE decreases from 1.004 ton/ha to 0.888 ton/ha. RF2, the yield estimation model based on remote sensing values alone has the lowest accuracy, probably because the amount of data input is far less than that of the other two models, and the quality of the data is greatly influenced by the scale effect. RF3 proved the feasibility of estimating yield with optimal FVC curve.

## Discussion

### Yield Estimation Benefits From Assimilating Fractional Vegetation Cover

From the results, the addition of assimilation only nets a slight improvement to the yield estimation accuracy, which may lead to doubts about the method effectiveness. Therefore, we establish the following assumptions to explain the benefit of the assimilation algorithm on yield estimation accuracy:

First, assume that Fs(t) is the FVC simulated by the AquaCrop model at time t, Fo(t) is the FVC observed by remote sensing at time t, and Ft(t) is the real FVC, which is unknown. Then, there are two errors, observation error _o_ and simulation errors _s_:


(13) $$\varepsilon_{\text{o}}={\text{F}}_{\text{o}}\left(\text{t}\right)-{\text{F}}_{\text{t}}\left(t\right)$$

(14) $$\varepsilon_{\text{s}}={\text{F}}_{\text{s}}\left(\text{t}\right)-{\text{F}}_{\text{t}}\left(t\right)$$

Furthermore, assuming that the error of observation value and the simulation value are unbiased and have no correlation:


(15) $$\text{E}\left(\varepsilon_{\text{o}}\right)=\text{E}\left(\varepsilon_{\text{s}}\right)=0$$

(16) $$\text{E}\left(\varepsilon_{\text{o}}\varepsilon_{\text{s}}\right)=0$$

At this time, by calculating the variance of the observation error σo2 and variance of simulation error σs2 and determining the corresponding weight coefficient, we obtained an FVC estimation value F_a_(t) that is closer to Ft(t). The reciprocal of variance is defined as the data precision.


(17) $${\text{F}}_{\text{a}}\left(\text{t}\right)={\text{aF}}_{\text{o}}\left(\text{t}\right)+{\text{bF}}_{\text{s}}\left(\text{t}\right)$$

Assuming that the estimated value is unbiased:


(18) $$\text{E}\left({\text{F}}_{\text{a}}\right)=\text{E}\left({\text{F}}_{\text{t}}\right)$$

(19) a+b=1

Then, minimize the variance σa2 of the estimated value F_a_(t):


(20) σa2=E[Fa(t)-Ft(t)]2=E(aFo(t)-Ft(t))+(bFs(t)-Ft(t))2

(21) =E[(aεo+bεs)2]=a2σo2+b2σs2

(22) a=σs2σo2+σs2

(23) b=σo2+o2σs2

(24) σa2=σo21+(σoσs)2<σo2

(25) σa2=σs21+(σsσo)2<σs2

Obviously, the variance of the estimated value is always less than the observation error variance and simulation error variance. The coupling of observation and model simulation information is confirmed to obtain a better state estimation value.

However, no data source can guarantee the unbiased of its error nature. In terms of remote sensing data, if the observations are collectively higher than the true values, the addition of observed values during assimilation will lead the simulation process to a higher direction but make the estimation results more distorted. Therefore, it is necessary to control and reduce the reliability of each input data of the assimilation algorithm to ensure the final prediction effect. This requires the continued development of remote sensing technology and traditional mechanism models.

### Uncertainties and Future Work

This paper mainly has uncertainty and limitations in the following two aspects: data assimilation by remote sensing and machine learning. In the ideal case, the AquaCrop model assimilation system coupled with multitemporal remote sensing products integrates the advantages of the two. On the one hand, the addition of a mechanism model makes up for the lack of biophysical significance of the system. On the other hand, the addition of remote sensing observations improves the reliability of the system and expands the application scope. But in fact, the analog of state variables in the crop model is not detailed enough, and many variation characteristics of FVC observed by remote sensing are not reflected in the simulation results. It should also focus on developing regional crop models to allow the input and output of surface parameters to better integrate and utilize regional products and serve regional research. In regional studies, remote sensing data are generally expected to be used due to their large scale and ready availability. However, the unverifiable authenticity of remote sensing data caused by mixed pixels must not be overlooked, and the final error may be three times the input LAI error (Fang et al., 2018). Although high spatial resolution imagery ensures the maximum number of pure pixels, the time resolution and width are sacrificed, resulting in high costs in regional applications. Research should balance the system investment and its efficiency. Above all, deliberately combining the advantages of different data resources to improve the practical value of crop models is still a valuable problem for agricultural research.

Introducing the data-driven method, this study established the regression relationship between time series FVC and yield based on the machine learning method of random forest. According to the crop yield predicted by the assimilated FVC and the statistical yield, the R^2^ of random forest reached 0.557, demonstrating that the construction logic of the system is tenable. It revealed that crop yield is not only reflected by FVC, which may account for 55.7% of the representation. The maize yield estimation accuracy was limited by the correlation between FVC and maize yield itself, which can hardly be surpassed by technology. For regional yield estimation, this is still a rare attempt to provide a relatively accurate yield forecast based only on time series FVC data.

Several previous studies have also found the significant association between surface state variables and crop yield, and all committed to estimating yield through these variables. Present yield estimation accuracy of 0.888 ton/ha through FVC assimilation has an advantage over previous assimilation studies. Hank et al., 2015) estimated winter wheat yield by assimilating LAI into PROMET model, the R^2^ was 0.93 and the RMSE was 1.15 ton/ha. Gilardelli et al. (2019) estimated rice yield in northern Italy by assimilating LAI into WARM rice model, the MAE was 0.66 ton/ha and the RRMSE was 13.8%. Their study got higher accuracy by using fine spatial resolution (30 meters) on an area lower than 3 km^2^, while our study using 500 m resolution product so as to achieved 20 km^2^ of yield estimation. Li et al., 2019) estimated winter wheat yield by assimilating LAI into WheatSM model, the RMSE was 1.641 ton/ha through SCE-UA assimilation method and 1.587 ton/ha through EnKF assimilation method. Wang P. X. et al. (2020) integrated remote sensing LAI of 4 phases to CERES-Maize model based on EnKF data assimilation approach in North China, the R^2^ of the estimated yield was 0.33, and the root mean square error (RMSE) was 0.371 ton/ha. Their study has only been verified in 8 research points for 5 years, while our study verified on 155 points for 21 years.

The results of this paper also show that there are many potential development space for the method of yield estimation based on the assimilation of surface state variable and machine learning. This finding is similar to those reported by Silva et al. (2020), which concluded that “big data” are useful to characterize cropping systems at the regional scale but need more progress to explain yield variability. Directions for improvement including optimizing machine learning algorithms to isolate and enhance the effect of FVC features on crop yield; and exploring multivariable joint assimilation integrating relevant biophysical indicators, such as evapotranspiration and temperature. It may also be necessary to apply continuous assimilation of multiple images into crop models to retain their spatial information.

## Conclusion

This study proposed a yield prediction method based on a crop model and remote sensing data assimilation for maize in the black soil region of Northeast China. The calibrated AquaCrop model already has a good simulation effect at the point scale, which confirms the availability of the AquaCrop model in this area. Profit from the physiological response of crops to environmental and management conditions is intuitively reflected by the AquaCrop model. We applied the model to simulate the growth of maize crops in the Songnen black soil area from 2000 to 2020 and accumulated a large database. After confirming the accuracy of the remote sensing surface parameter products, synchronous time series observations are added to the AquaCrop simulation results of 21 years through the EnKF filtering assimilation algorithm, and an optimal FVC dataset is established. Using the optimal FVC and the regression relationship between FVC and statistical yield trained by random forest, a yield estimation method is formed. Data assimilation combines the two geodetic research methodologies of simulation and observation, while this study proposed that the method further integrates the idea of a mechanism model and machine learning and provides a feasible idea for crop yield estimation. Overall, the main contribution of current study is offering new insights and perspectives for the following two issues: one is how to integrate satellite remote sensing data into the crop model at the regional scale; the second is how to obtain more useful yield information from the available surface parameter data.

## Data Availability Statement

The raw data supporting the conclusions of this article will be made available by the authors, without undue reservation.

## Author Contributions

YC: conceptualization, methodology, software, formal analysis, investigation, and writing—review and editing. SL: conceptualization, writing—review and editing, supervision, and project administration. XL and HG: visualization. YX: data curation, conceptualization, and supervision. YH: data curation. All authors have read and agreed to the published version of the manuscript.

## Conflict of Interest

The authors declare that the research was conducted in the absence of any commercial or financial relationships that could be construed as a potential conflict of interest.

## Publisher’s Note

All claims expressed in this article are solely those of the authors and do not necessarily represent those of their affiliated organizations, or those of the publisher, the editors and the reviewers. Any product that may be evaluated in this article, or claim that may be made by its manufacturer, is not guaranteed or endorsed by the publisher.

## Figures and Tables

**FIGURE A1:**
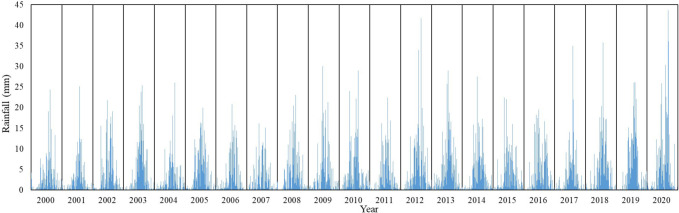
Average daily precipitation in Songnen Plain from 2000 to 2020.

**FIGURE A2:**
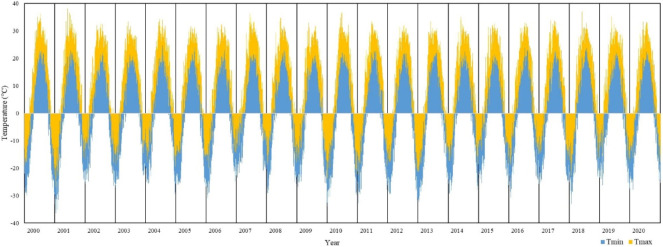
The average daily temperature in the Songnen Plain from 2000 to 2020 is close to the average long-term situation. The gentle temperature change provides a better planting environment and is conducive for crops to adapt to environmental changes.

**FIGURE A3:**
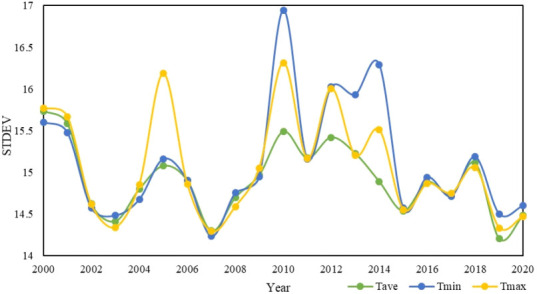
Standard deviation of annual average temperature in Songnen Plain from 2000 to 2020.

**FIGURE A4:**
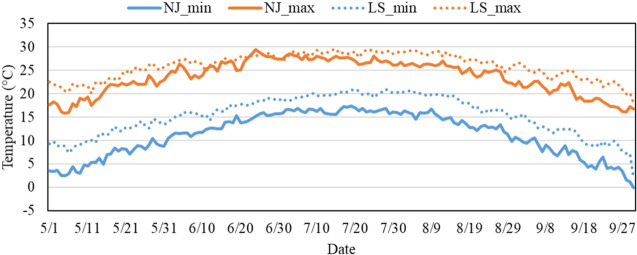
Average temperature of the northern and southern parts of the Songnen black soil region during the recent 21-maize growth season.

## Footnotes

1
http://data.cma.cn/site/index.html

2
http://www.cresda.com/CN/Data

3
http://www.geodata.cn/thematicView/GLASS.html

## Appendix

### Appendix A: Meteorological Data

**TABLE A1:** Weather station information in Songnen Plain.

Station ID	Latitude	Longitude	Altitude (m)	Province	County
50557	49.17	125.23	242.2	Heilongjiang	Nenjiang
50639	48.00	122.73	306.5	Inner Mongolia	Zhalantun
50656	48.28	126.52	269.7	Heilongjiang	Beian
50658	48.05	125.88	236.0	Heilongjiang	Keshan
50674	48.05	125.88	236.0	Heilongjiang	Keshan
50739	47.33	123.18	190.0	Heilongjiang	Longjiang
50742	47.80	124.48	162.7	Heilongjiang	Fuyu
50745	47.38	123.92	147.1	Heilongjiang	Qiqihaer
50756	47.45	126.97	239.2	Heilongjiang	Hailun
50758	47.18	125.90	246.0	Heilongjiang	Mingshui
50844	46.40	123.45	138.8	Heilongjiang	Tailai
50853	46.62	126.97	179.6	Heilongjiang	Beilin
50854	46.38	125.32	149.3	Heilongjiang	Anda
50862	46.98	128.02	210.5	Heilongjiang	Tieli
50936	45.63	122.83	155.3	Jilin	Baicheng
50945	45.50	124.27	137.6	Jilin	Da’an
50948	45.00	124.02	146.3	Jilin	Qian’an
50949	45.08	124.87	136.2	Jilin	Qianguo
50950	45.70	125.25	148.7	Heilongjiang	Zhaozhou
50953	45.75	126.77	142.3	Heilongjiang	Harbin
50955	45.38	126.30	166.4	Heilongjiang	Shuangcheng
54041	44.80	123.07	150.0	Jilin	Tongyu
54049	44.25	123.97	188.9	Jilin	Changling
54063	44.97	126.00	196.8	Jilin	Fuyu
54064	44.38	125.15	170.2	Jilin	Nong’an
54142	43.50	123.53	114.9	Jilin	Shuangliao
54157	43.17	124.33	165.7	Jilin	Siping
54161	43.90	125.22	236.8	Jilin	Changchun
54165	43.55	125.63	219.5	Jilin	Shuangyang

**TABLE A2:** Statistics of annual precipitation on the Songnen Plain.

Year	2000	2001	2002	2003	2004	2005	2006
Sum	394.450	308.154	472.461	541.175	362.904	567.989	457.154
Std dev	2.171	1.991	2.547	2.668	2.056	2.530	2.368
Year	2007	2008	2009	2010	2011	2012	2013
Sum	360.679	491.032	492.386	524.946	449.171	619.689	646.086
Std dev	2.015	2.482	2.555	2.634	2.316	3.085	2.982
Year	2014	2015	2016	2017	2018	2019	2020
Sum	527.464	514.336	554.368	469.113	591.448	652.820	711.384
Std dev	2.501	2.365	2.539	2.784	2.885	3.003	3.915

*2020 has both the highest total precipitation and the highest standard deviation in the past two decades, which alerts for climate anomalies in the future, and abnormal precipitation will have a serious impact on agricultural production.*

### Appendix B: AquaCrop Model Parameters

**TABLE A1:** Parameter calibration of the AquaCrop model simulating the growth of maize in the Northeast Chinese black soil area.

Notation	Description	Unit	Default value	Calibration value
Pexp-lw	Soil water depletion threshold for canopy expansion – lower threshold	% of TAW	0.25	0.14
Pexshp	Shape factor for Water stress coefficient for canopy expansion	–	3.0	2.9
DeKcTr,x	Decline of crop coefficient as a result of aging, nitrogen deficiency	% d-1	0.150	0.300
KcTr,x	Crop coefficient before canopy formation and senescence	–	1.10	1.05
Rexshp	Shape factor describing root zone expansion	–	1.5	1.3
Zx	Maximum effective rooting depth	m	1.0	2.3
WP*	Water productivity normalized for ET0 and CO2	g m-2	17.0	33.7
CCx	Maximum canopy cover	%	80	96
CGC	Canopy growth coefficient	% d-1	15.0	15.8
CDC	Canopy decline coefficient	% d-1	12.8	11.7
HI0	Reference harvest index	%	50	58

*TAW: total available water in the root zone.*
